# Characteristics and Frequency of Pediatric Inflammatory Bowel Disease-Associated Pancreatitis: A Japanese Nationwide Survey

**DOI:** 10.1097/PG9.0000000000000162

**Published:** 2022-01-24

**Authors:** Kenji Hosoi, Kei Minowa, Mitsuyoshi Suzuki, Takahiro Kudo, Yoshikazu Ohtsuka, Takeshi Tomomasa, Hitoshi Tajiri, Takashi Ishige, Hiroyuki Yamada, Katsuhiro Arai, Atsushi Yoden, Kosuke Ushijima, Tomoki Aomatsu, Satoru Nagata, Keiichi Uchida, Kazuo Takeuchi, Toshiaki Shimizu

**Affiliations:** From the * Department of Pediatrics, Juntendo University Faculty of Medicine, Tokyo, Japan; † Division of Gastroenterology, Tokyo Metropolitan Children’s Medical Center, Tokyo, Japan; ‡ Members of the Japanese Society for Pediatric Inflammatory Bowel Disease Working Group; § PAL Children’s Clinic, Gunma, Japan; ∥ Department of Pediatrics, Kinki University Faculty of Medicine, Osaka, Japan; ¶ Department of Pediatrics, Gunma University Graduate School of Medicine, Gunma, Japan; # Department of Pediatrics, Osaka Hospital, Japan Community of Healthcare Organization, Osaka, Japan; ** Division of Gastroenterology, National Center for Child Health and Development, Tokyo, Japan; †† Department of Pediatrics, Osaka Medical and Pharmaceutical University, Osaka, Japan; ‡‡ Department of Pediatrics, Dainikyoritsu Hospital, Hyogo, Japan; §§ Department of Pediatrics and Child Health, Kurume University, Fukuoka, Japan; ∥∥ Department of Pediatrics, Tokyo Women’s Medical University Hospital, Tokyo, Japan; ¶¶ Department of Gastrointestinal and Pediatric Surgery, Mie University Graduate School of Medicine, Mie, Japan; ## General Health Support Center, Gunma University Graduate School of Medicine, Gunma, Japan.

**Keywords:** Crohn disease, ulcerative colitis, extraintestinal manifestations, autoimmune pancreatitis

## Abstract

Acute pancreatitis (AP) develops in approximately 2% of patients with the diagnosis of inflammatory bowel disease (IBD), but the characteristics and frequency of childhood-onset IBD-associated AP in Japan have not been studied. The present study aimed to clarify the characteristics of IBD-associated AP in Japan.

**Methods:**

A nationwide survey of pediatric patients with IBD (age, <17 years) was conducted from December 2012 to March 2013 at 683 hospitals and medical centers in Japan. A secondary survey was also sent to the centers with the target patients to evaluate their characteristics.

**Results:**

The response rate to the first part of the survey was 61.2% (n = 418). In total, 871 patients with Crohn disease and 1671 patients with ulcerative colitis were enrolled. The second part of the survey found that 11 (1.3%) patients with Crohn disease and 23 (1.4%) patients with ulcerative colitis experienced IBD-associated AP caused by medication (n = 18, 53%), a primary disease (n = 11, 32%), autoimmune pancreatitis (n = 1, 3%), or an anatomical abnormality (n = 1, 3%). All the patients had only mild AP.

**Conclusions:**

IBD-associated AP was not very frequent and was generally mild. The major cause of the pancreatitis was the medication used to treat the IBD.

**What Is Known**

- The incidence of inflammatory bowel disease (IBD)–associated acute pancreatitis (AP) in pediatric patients was reportedly about 2% although only a few studies have examined this topic.
- The most common causes of IBD-associated AP in pediatric patients are drugs, duodenal involvement, and hepatobiliary complications.

**What Is New**

- This study was the first to examine IBD-associated pancreatitis in Japanese children and found its incidence in the target population to be 1.4%.
- Although severe cases have been reported in the past, in the present study, all IBD-associated AP cases in Japanese children were mild.

## INTRODUCTION

Inflammatory bowel disease (IBD) includes chronic, relapsing, inflammatory conditions of the gastrointestinal tract, including ulcerative colitis (UC), Crohn disease (CD), and IBD unclassified.

IBD can cause extraintestinal manifestations (EIMs) involving the joints, skin, bone, liver, eyes, or pancreas. EIMs reportedly develop in 6% to 47% of adult patients and 16.7% to 68% of pediatric patients (1–7). Acute pancreatitis (AP) is one of the important complications of IBD, and its incidence in patients with IBD is commonly thought to be much higher than in the general population. A previous case series reported a higher incidence of AP in patients with CD than in those with UC (8). Whereas approximately 25% of AP cases in the general population are severe and require intensive care, a previous report demonstrated that AP in CD may be self-limited (9). However, few epidemiological data on pediatric IBD-associated AP are available. Thus, the present study aimed to clarify the characteristics of pediatric IBD-associated AP in Japan.

## METHODS

### Materials and Methods

A 2-part postal survey was conducted. The goal of the first part was to evaluate the number of pediatric patients with IBD and IBD-associated AP. The second part aimed to obtain clinical information. The protocol of this study complies with the ethical guidelines of the Declaration of Helsinki and was approved by the Institutional Ethics Committee at Juntendo University (number 12-099).

### First Survey

The target subjects were patients with IBD and IBD-associated AP who were seen at the participating centers between 2000 and 2012. The centers surveyed were chosen from among Board-Certified Pediatrician Training Center and Board Pediatric Surgeon Training Center members participating in the Inflammatory Bowel Diseases Study Group of the Ministry of Health and Welfare of Japan. The questionnaire was sent to 683 centers between December 2012 and March 2013. IBD was diagnosed by each physician using the Japanese IBD criteria (10), which are mostly identical to the Port criteria (11). AP was diagnosed using the diagnostic criteria of the Japanese guidelines for AP management (12). Patients with IBD meeting any two of the following criteria received the diagnosis of IBD-associated AP: (1) abdominal pain compatible with AP; (2) serum amylase and/or lipase levels >3 times the upper limit of normal; and (3) imaging findings of AP.

### Second Survey

Detailed clinical data on age, sex, age at disease onset, and the severity and clinical course of IBD-associated AP were collected.

## RESULTS

### First Survey

In total, 418 institutions responded (response rate, 61.2%; 418/683) to part one of the survey. Table 1 shows the results. A total of 2488 pediatric patients with IBD (871 with CD and 1617 with UC) were enrolled between January 1, 2000, and December 31, 2012, including 34 patients with IBD-associated AP (1.3% [11/871] of the patients with CD and 1.4% [23/1617] of the patients with UC). The male-to-female ratio of the CD and UC groups with IBD-associated AP was 2.7 (8/3) and 1.1 (12/11), respectively. Age at IBD-associated AP onset in the CD and UC groups and in the entire IBD cohort was 13 years, 13 years, and 12 years, while the median age of IBD-associated AP onset was 13 years 5 months, 13 years 4 months, and 12 years 6 months, respectively.

**TABLE 1.** Baseline characteristics of pediatric IBD-associated AP cases

	Crohn disease	Ulcerative colitis
No. of patients	871	1617
No. of patients with IBD-associated AP	11 (1.3%)	23 (1.4%)
Sex (male/female) of patients with IBD-associated AP	2.7 (8/3)	1.1 (12/11)
Median age at IBD-associated AP onset	13 years 5 months (range, 8 years 3 months to 15 years 4 months)	13 years 4 months (range, 9 years 4 months to 18 years 1 month)

AP = acute pancreatitis; IBD = inflammatory bowel disease.

### Second Survey

In patients with IBD-associated AP, 76% had abnormal imaging findings, and all the patients had abdominal pain and elevated pancreatic enzymes (Table 2). The etiology of the IBD-associated AP was IBD medication in 53% (18/34), a primary disease in 32% (11/34, 4 CD and 7 UC patients), an autoimmune response in 3% (1/34), an anatomical abnormality in 3% (1/34), and other in 9% (3/34) of the cases.

**TABLE 2.** Demographic and clinical data of IBD-associated AP cases

	IBD-associated AP
Etiology	
Original disease	32% (11/34, 4 CD and 7 UC)
Medication for IBD	53% (18/34)
	5-ASA: 8
	AZA/6-MP: 9
	IFX: 1
	CYA: 1
	Others: 1
Autoantibody	3% (1/34)
Anatomic abnormality	3% (1/34)
Unknown	9% (3/34)
Time of onset	
Before remission	44% (15/34)
At remission	26% (9/34)
At relapse	29% (10/34)
Severity score	0: 94% (29/32)
	1: 3% (1/32)
	2: 3% (1/32)
	3: 0%
Treatment	Fasting: 34
	Protease inhibitor: 23
	Antiacid: 19
	Antibiotic: 12
	Ulinastatin: 4
	Predonisolone: 1
	Plasma exchange: 1
Course	Remission: 88% (28/32)
	Recurrence: 9% (3/32)
	Chronicity: 3% (1/32)

5-ASA = 5-aminosalicylic acid; 6-MP = 6 mercaptopurine; AP = acute pancreatitis; AZA = azathioprine; CD = Crohn disease; CYA = cyclosporine; IBD = inflammatory bowel disease; IFX = infliximab; UC = ulcerative colitis.

The drug-induced pancreatitis cases in the present study were caused by 5-aminosalicylic acid in 8 cases, azathioprine (AZA) or 6-mercaptopurine in 9 cases, infliximab in 1 case, and cyclosporine in 1 case. Of the AP cases following AZA treatment, the duration from the start of medication to disease onset was 2 to 4 weeks. IBD-associated AP occurred before the diagnosis in 44% (15/34 cases), in the remission phase in 26% (9/34 cases), and at relapse of the disease in 29% (10/34 cases) of the cases. The severity of AP was evaluated using the pediatric Japan scoring system (severe was defined as >3 points) (13). In the present study, 94% (29/32) of patients had a score of 0, 3% (1/32) had a score of 1, and 3% (1/32) had a score of 2, indicating that all the cases were mild. The treatments for IBD-associated AP were fasting (n = 34), protease inhibitor (n = 23), gastric antacid (n = 19), antibiotics (n = 12), ulinastatin (n = 4), prednisolone (n = 1), and plasma exchange (n = 1). In all the patients with the diagnosis of drug-induced AP, the drug was discontinued. The outcomes of the IBD-associated AP were remission in 83%, recurrence in 9%, and chronic disease in 3% of the cases. Regarding the clinical course of the patients, 73% of the patients with IBD-associated AP experienced AP during the active phase of IBD, and 32% of those with the disease improved when their IBD symptoms resolved.

## DISCUSSION

The present study enrolled 871 patients with CD and 1617 patients with UC. Almost 1% to 2% of the CD and UC groups had nearly the same rate of IBD-associated AP development. The major etiology of IBD-AP was IBD medication, and almost half the causative drugs were AZA or 6-mercaptopurine. In most cases, the onset of IBD-associated AP occurred during the active phase of IBD and was mild in all the cases.

IBD-associated AP is considered a rare form of EIM, but it can occur more often in patients with IBD than in the general population (14). Recent studies have demonstrated that the incidence of IBD-associated AP ranged from 1.4% to 1.6% in adults (15,16). On the contrary, the incidence of IBD-associated AP in pediatric patients was reportedly 2.2% (17) although only a few studies have been published on this topic (18,19). The incidence of IBD-associated AP in the present study was comparable to that in previous reports of adult and pediatric IBD patients. Furthermore, in a comparison of UC and CD in adult patients, IBD-associated AP was more common in patients with CD than in those with UC (20), whereas no difference was observed among pediatric patients (21). In the present study, the AP incidence was comparable between the CD and UC groups. Since IBD-associated AP often presents with abdominal pain, the most common symptom of IBD, it may be overlooked as a flare-up of IBD. The disease may also be misdiagnosed because of its mild severity; thus, the actual number of patients may be even larger than currently known.

Factors leading to the development of AP include drugs, duodenal lesions in IBD, hepatobiliary diseases (gallstones, primary sclerosing cholangitis, etc), granulomatous lesions in the ampulla of Vater or bile duct, and autoimmune disorders. The most common causes of IBD-associated AP are gallstones and drugs in adults (22) although a retrospective study of 124 pediatric patients with IBD reported that the cause of IBD-associated AP was medication in 25%, duodenal involvement in CD in 18%, and hepatobiliary complications in 15% of the patients (18). The mechanism underlying drug-induced AP is unclear but may involve direct toxicity, hypersensitivity, secondary hyperlipidemia, or hypercalcemia. Previous reports demonstrated that 5-aminosalicylic acid, AZA, 6-MP, and corticosteroids induced pancreatitis (23–25), while AZA and 6-MP were associated with a high risk of IBD-associated AP within 3 weeks after starting treatment (26), a result in line with the findings of the present study. Drug-induced pancreatitis was the most common cause of IBD-associated AP in the present study, with most of the cases being caused by thiopurine (23.5%) or aminosalicylic acid preparations (26.5%). These results were in line with those reported for adult IBD-associated AP (4). Aminosalicylic acid preparations are necessary to induce and maintain remission in pediatric IBD, and thiopurine preparations are commonly used as maintenance therapy in pediatric patients with moderate-to-severe IBD. Therefore, due consideration should be paid to the potential for drug-induced pancreatitis in pediatric patients with IBD. Steroids, which are frequently used during the acute phase of IBD, can also induce pancreatitis, but the causal relationship remains controversial (23).

An Italian cohort study reported that all 27 cases of pediatric IBD-associated AP analyzed were mild and resolved spontaneously (21) as in the present study. Generally, AP is thought to be a severe disease, but most cases of IBD-associated AP are drug induced, and the symptoms usually improve with drug discontinuation alone. However, there is a report of a severe case of IBD-associated AP with a heterozygous mutation of the *CFTR* gene (27). Thus, further study of the etiology of IBD-associated AP is needed.

A previous study of the timing of AP onset reported that IBD-associated AP was more frequent during the active phase of IBD (22) as borne out by the present study. This finding indicates that systemic inflammation may be one of the factors in AP development.

The present study was the first to examine IBD-associated pancreatitis in Japanese children. This was a retrospective study and has several limitations. First, abdominal pain due to AP may have been underestimated because it may have been diagnosed as a flare-up of IBD itself. Also, mild cases may have been overlooked. Second, there was no comparison with healthy subjects. Third, although an association between disease extent and AP onset has been reported, disease extent in CD or UC was not evaluated in the present study.

In conclusion, the incidence of IBD-associated pancreatitis is not very high. The main cause of the disease is the medication used to treat the IBD itself. Most cases of IBD-associated AP are mild, but severe cases have also been reported. The possibility of AP should be considered whenever gastrointestinal symptoms appear during the course of IBD.

## ACKNOWLEDGMENTS

The authors would like to thank the members of the Ministry of Health, Labour and Welfare of Japan’s Inflammatory Bowel Diseases Study Group, especially the Department of Gastroenterology and Hepatology at Tokyo Medical and Dental University (Mamoru Watanabe), the Department of Internal Medicine at Sakura Medical Center, Toho University (Yasuo Suzuki), the Department of Internal Medicine at the National Defense Medical College (Soichiro Miura and Ryota Hokari), and the Center for Advanced IBD Research and Treatment at Kitasato Institute Hospital, Kitasato University (Toshifumi Hibi), for their contributions to this work.
